# Do Not Go Gentle Into That Good Night

**DOI:** 10.14797/mdcvj.1079

**Published:** 2022-03-14

**Authors:** James B. Young

**Affiliations:** 1 Executive Director of Academic Affairs, Cleveland Clinic, Professor of Medicine, Cleveland Clinic Lerner College of Medicine, a program of Case Western Reserve University School of Medicine, Cleveland, Ohio, US

## Abstract

Dylan Thomas (1914–1953) was a Welsh poet and screenwriter, whose poems are lyrical, with entertaining wordplay, vivid imagery, and captivating rhythms. He may be best known for his poem “Do Not Go Gentle Into That Good Night,” contained in *The Poems of Dylan Thomas* (New Directions Publishing, 1971). The poem’s imagery of the closing day and coming nightfall allude to the cyclical process of life and death.

**Publisher’s Note:** A correction article relating to this paper has been published and can be found at https://journal.houstonmethodist.org/articles/10.14797/mdcvj.1621.

## Some Poems must be Read Aloud

Dylan Thomas was born in Wales, United Kingdom, in 1914 and died much too young in 1953. He was in New York City on his fourth trip to America. One of the better-known 20th century poets, Thomas is often pigeonholed as a Romantic or Post-Romantic. He didn’t generally mix with those in the Imagist movement (such as William Carlos Williams) or the Beats (such as Alan Ginsburg), many of whom were his contemporaries, but he could be characterized as being in their periphery because of his love of wordplay.

Thomas drank to excess and probably enjoyed a drug or two. His life was hard scrabble: he was peripatetic and always short of cash, even as he was becoming a recognized writer and poet. Words and writing were his passion from a young age. In fact, he was drawn to literature through nursery rhymes that he heard as an infant; he came to understand those words even before he could read, which isn’t surprising given that his father was a teacher with an English literature degree from University College, Aberystwyth. But Thomas wasn’t only a poet. He also wrote short stories and film scripts, with many of his works published when he was just a teenager.

During World War II, Thomas was able to avoid combat due to a medical condition characterized as an “unreliable” lung. Instead, he wrote several screenplays for the British Broadcasting System while continuing to drink excessively. One film of interest to us healthcare providers is *Conquest of a Germ* (1944), dedicated to the researchers and doctors who discovered sulfonamides, which at the time revolutionized treatment of many infectious diseases and preceded penicillin.

Unlike his screenplays, Thomas’ poems are lyrical mashups with plentiful entertaining wordplay. *Do Not Go Gentle Into That Good Night* is a typical example, capturing you with its images, rhythms, and rhymes. It is best read aloud to savor the wordplay and reveal a deeper meaning. Unlike a sonnet, this writing is a villanelle—a 19-line poem with five stanzas comprised of three lines each and concluding with a quatrain (a four-line phrase). Two rhymes are used repeatedly throughout the poem: “Rage, rage against the dying of the light” and “Do not go gentle into that good night.” Searching for deeper allegory, it helps to know that Thomas’ father was said to be gravely ill when the poem was crafted (1947). The reference to a closing of the day and coming nightfall seems related to the graying pall of a dying father, with an admonition to struggle against a seeming fate.

**Figure:**
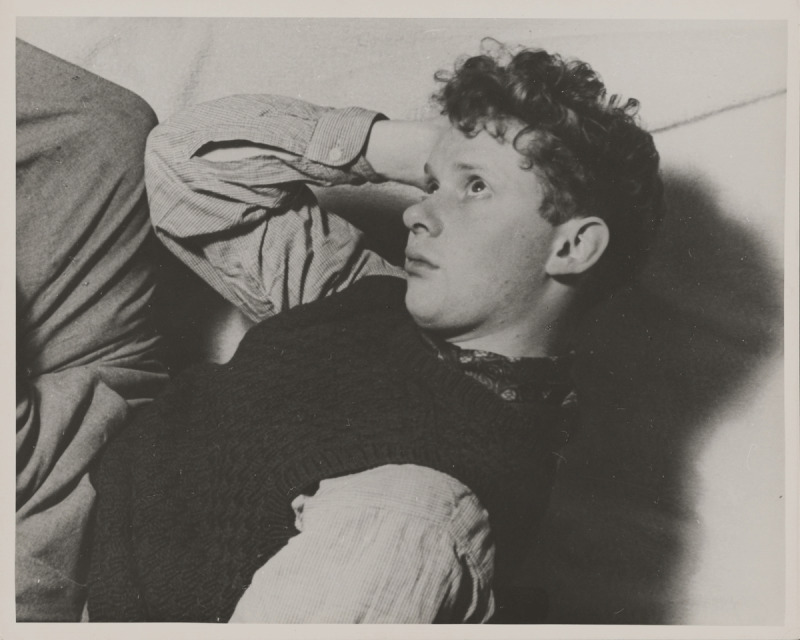
Photograph of Dylan Thomas. Unidentified photographer, undated. Dylan Thomas Collection, Harry Ransom Center, via Radio Times Hulton Picture Library and the Imperial War Museum.

As we continue to struggle with today’s pandemic and the toll it has taken, Thomas’ poem encourages us to think about the cyclical process of life and death, especially our efforts to staunch premature death while recognizing when dying becomes an unavoidable passage that should be nurtured. Dylan Thomas’ death was not a peaceful one. He raged against a setting sun and the dying of light. Continuing to drink right up to the end, he had been given narcotics in the setting of what might have been pneumonia, sepsis, and an alcohol-induced encephalopathy. Just after turning 39 years old, after a day at a favorite New York pub, he fell into a coma from which he never awoke. His poetry lives on for us to study, reflect on, and enjoy.

