# Correction: Do Not Go Gentle Into That Good Night

**DOI:** 10.14797/mdcvj.1621

**Published:** 2025-05-12

**Authors:** James B. Young, LaVonne Carlson

**Affiliations:** 1 Cleveland Clinic, Professor of Medicine, Cleveland Clinic Lerner College of Medicine, a program of Case Western Reserve University School of Medicine, Cleveland, Ohio, US

## Abstract

This article details a correction to: Do Not Go Gentle Into That Good Night. Methodist DeBakey Cardiovasc J. 2022; 18(2): 111-113. doi: 10.14797/mdcvj.1079

## Correction

This corrects the article “Do Not Go Gentle Into That Good Night” in Volume 18, Issue 2, page 111,^1^ with a revised Abstract. The previous version contained incorrect information, which was withdrawn for legal reasons. The Estate of Dylan Thomas has confirmed that the poem remains in copyright in the United States of America.
